# Causal and Predictive Data Analysis for Conservation: Simulation Based Comparisons and a Case Study for Detecting Impact of Artificial Feeding on Eurasian Red Squirrel ( *Sciurus vulgaris* )

**DOI:** 10.1002/ece3.72142

**Published:** 2025-09-16

**Authors:** Yusaku Ohkubo, Ozora Takeda, Kenta Uchida

**Affiliations:** ^1^ Okayama University Okayama Japan; ^2^ The Institute of Statistical Mathematics Tokyo Japan; ^3^ Graduate School of Agriculture and Life Science The University of Tokyo Tokyo Japan

**Keywords:** artificial feeding, causation, model selection, propensity score, statistics

## Abstract

Estimating the causal effect of a variable is an important task for applied ecology. While several methods have been applied to empirical, observational studies, there have been a few attempts to employ the causal inference approach based on the propensity score methods in ecology and evolutionary biology despite its widespread usage in other scientific fields. This paper applies the overlapping‐weighted estimator to the Eurasian red squirrel *Sciurus vulgaris* to evaluate human activity on behavioral tolerance to humans as a model case. This statistical method is one of the common propensity score methods in the statistical community to better evaluate the causal effect of particular variables on a target variable. We focused on the effect of artificial feeding on tolerance to humans because feeding has been suggested to be a main driver of habituation to humans, while the causal effect has not been statistically tested. We performed an estimation of causal effects and compared results with the analysis that employed commonly used methods including AIC and LASSO. The results showed that the effect of artificial feeding is larger than previously known and that AIC and LASSO yielded biased results by dismissing confounding variables. Our results indicate that propensity score methods can be useful for wildlife management by offering a more accurate evaluation of causal effects.

## Graphical abstract

We applied propensity score methods to evaluate the causal effect of artificial feeding on wildlife tolerance to humans, using both simulation and a real‐world case study of Eurasian red squirrels. Our findings demonstrate that predictive methods like AIC and LASSO may underestimate such effects, while causal inference provides more accurate and management‐relevant estimates. This study highlights the utility of causal approaches for informing conservation and urban wildlife management decisions.

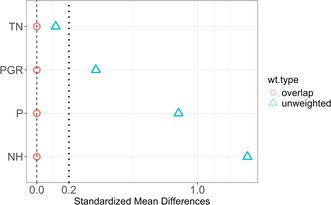

## 1 Introduction

The biggest challenge in ecology is, arguably, to disentangle the complexity of organism‐environmental interactions. In particular, ecologists attempt to explain causal relationships between variables using a variety of methods. Although one of the traditional and fundamental approaches is a randomized controlled trial (RCT) that tests a causal effect between variables, where a subject (i.e., a unit of observation) is assigned to a controlled or a treatment group at random before the data is collected, this is not a realistic option in most of the empirical studies (Christie et al. 2019). Particularly, for field‐based observational studies, human resources, budgets, or ethical concerns might restrict the data collection design. However, as the importance of conservation ecology grows, exploring causal relationships between ecological drivers and consequences has become increasingly important. Detecting key factors that influence target ecological consequences (e.g., behavior, population demography, species richness, and biodiversity) is crucial for suggesting concrete and effective management actions for biodiversity conservation. It is therefore a clear challenge for ecologists and statisticians to develop practical statistical approaches for examining causal relationships between variables.

Statistical data analysis based on predictive approaches has long been the most common alternative among ecologists (Burnham and Anderson 2001; Aho et al. 2014). For example, Akaike information criteria (AIC) (Akaike 1974)—a statistical method to detect the “important” variables by comparing different combinations of explanatory variables in multiple models is—widely used for variable selection of multiple regression models. Penalized‐likelihood (also known as “regularizing”) methods including Ridge and LASSO are also emerging methods that have attracted researchers because they handle larger and/or higher‐dimensional datasets (Morii et al. 2018). These methods solve both parameter estimation and variable selection problems simultaneously with a lower computational cost than exploring all the combinations of explanatory variables using AIC. These methods, however, are not always satisfactory because they often lack the theoretical justification for causality. Although information criteria and the penalized‐likelihood methods are common, these methods aim to find the best regression model for the prediction of the objective variable, not for the test of the causality between the objective variable and an explanatory variable, at least in a naïve form. Bringing causal insights into statistical analysis is desired, rather than predictive approaches.

Currently, the structural equitation model (SEM) approach has been gaining popularity (Lefcheck 2016; Fan et al. 2016; Shipley and Douma 2020) because it explicitly incorporates causality. This approach is a strong tool in cases where a dataset consists of a large number of variables, and also when we are interested in its “network” structure. Although SEM is a useful statistical tool for applied ecology, there are still several limitations. First, SEM requires a larger dataset. Since SEM consists of a set of “equations” that describe causal relationships between causes and their effects, the number of model parameters will be larger, lowering statistical efficiency. Second, SEM needs to be a fully specified parametric model. In other words, we need detailed assumptions on which variables cause which effects. Even when we are interested in the causality of only one particular explanatory variable while other explanatory variables are also needed to adjust confounding factors, we have to designate all the “network” structures of variables. These can also pose challenges in applied ecology. For example, evaluating a causal effect of a particular action and a policy might be of particular interest, while other environmental factors that are used as the controlling factors are less important to judging the effectiveness of the actions and policies. Specifying complete causal relationships between all the variables might be too demanding or risk misleading conclusions due to model misspecification. As such, results of SEM do not always directly translate into conservation actions. In order to propose realistic actions for management agencies, we need to differentiate which variables are controllable and which are not in the models. For these reasons, a more efficient and pragmatic methodology with robustness to model misspecification is helpful for causal inference.

This study aims to apply an alternative causal inference approach, rather than SEM, based on the propensity score methods and to compare its performance with predictive approaches of statistical analysis, including AIC‐based variable selection and LASSO estimator, which is popular among ecologists. The propensity score causal inference is a methodology developed in a seminal paper by Rosenbaum and Rubin (1983) within a framework called potential outcome. They considered a case when a cause variable *Z* is binary (= 1 if a treatment is introduced and = 0 otherwise) and defined the “causal effect” as the difference of a counterfactual comparison (see following section for details). Statisticians have proposed various estimators for this causal effect (e.g., matching estimator, weighted estimator, and covariate adjusting estimator) and showed that it can be consistently estimated. The advantage of the propensity score approach is that it requires a smaller sample size than SEM and is a semiparametric methodology, which does not require a fully parametric model and is thus robust to model misspecification (e.g., Drake 1993). These methods are now considered a defacto standard to evaluate causal effects in many scientific fields, including epidemiology (Joffe and Rosenbaum 1999), sociology (Thoemmes and Kim 2011), and economics (Dehejia and Wahba 1999). Despite the widespread usage in these fields, very few studies have attempted to apply the propensity score methods in ecological studies. Thus, the performance and applicability of propensity score methods have not been well tested (but see Ramsey et al. 2019; Kluender et al. 2024 for seminal cases). Although Ramsey et al. (2019) evaluated the performance of the propensity score methods via a simulation study and applied them to empirical data, the comparison with predictive approaches of statistical analysis has not been reported as far as we know.

In this study, we compared statistical performances of propensity score methods with predictive AIC and LASSO approach and applied these methods to test how a particular ecological driver (i.e., artificial feeding) influences wildlife's behavior (i.e., tolerance to human approach; details are below) using Eurasian red squirrel *Sciurus vulgaris* living in urban parks. Artificial feeding is widely observable direct human‐wildlife interaction (Cox and Gaston 2018) and has been known to be one of the strongest factors that modify a wide range of wildlife's behaviors, including increased tolerance to humans in many species (Møller et al. 2015; Uchida et al. 2021). While this feeding‐induced increased human tolerance may provide an opportunity for human‐nature interaction (Uchida et al. 2023) short‐term benefit in species, it could lead to the negative ecological consequences and human‐wildlife conflicts, such as increased predation risk (Shutt and Lees 2021), disease transmission and human injury (Uchida et al. 2023). Assessing how artificial feeding modifies wildlife's tolerance to humans is an important topic in wildlife conservation and management, especially to the species in urban areas. However, most prior studies have examined the effect of feeding on wildlife's tolerance to humans by using correlational approaches between antipredator behavior to human presence and the presence of feeding, and few studies have rigorously tested the causal effect.

In this article, we initially give a brief introduction to the propensity score causal inferential methodologies. Then, we apply one of these methods to simulated data and real data, which was originally analyzed by Uchida et al. (2021). We also compare the results with other commonly used predictive methods, including the variable selection based on AIC and the penalized‐likelihood LASSO estimator. We finally discuss the results and examine several implications for further applications of the methods.

## 2 Brief Introduction to the Propensity Score Causal Inference

This section gives a brief overview of the propensity score causal inference. We do not intend to give a complete review of the methodology nor the theoretical justifications. Rather, we introduce key ideas for empirical ecologists. For interested readers, see Austin (2011, 2014), Leite (2016), or Guo and Fraser (2014) for example.

Propensity score analysis typically assumes that a dataset consists of three types of variables, with a sample size N. $Z=\left(z_{1},z_{2},\ldots z_{N}\right)$ is a binary variable that indicates whether an i‐th observation is in the treatment group ($z_{i}=1$) or untreated ($z_{i}=0$). $Y=\left(y_{1},y_{2},\ldots y_{N}\right)$ is an outcome variable, which would typically serve as the dependent variable in a standard regression analysis. $X=\left(x_{1},x_{2},\ldots x_{N}\right)$ is a set of confounding factors that may influence both Z and Y.

The goal of propensity score analysis is to obtain an accurate estimate of the causal effect of Y on Z, where the definition of causation is introduced later. A naïve approach would be to regress ZonY but this would yield a biased estimate of the causal effect if confounding variables X are present. Propensity score analysis removes or reduces this bias by adjusting differences of the distribution of X between treated group and untreated group.

The propensity score $e_{i}$ for i‐th observation is defined as

(1) $$e_{i}=\mathrm{Pr}\left(z_{i}=1|x_{i}\right)$$

That is the probability of i‐th observation is in the treatment group given all the covariate factors $x_{i}$. The propensity score is typically estimated by the logistic regression model or nonparametric machine learning techniques (e.g., general additive model, Bayesian additive regression tree. Lee et al. 2010).

The basic idea behind the propensity score causal inference is a concept of causality named “potential outcome.” Suppose to test whether a fertilizer (Z) causes a larger yield (Y) in a farm crop, for example. Rosenbaum and Rubin (1983) considered a comparison with a counterfactual situation $y_{i}\left(z_{i}=1\right)$ versus $y_{i}\left(z_{i}=0\right)$, where the former indicates that the crop size of the subject *i* when received the treatment (i.e., fertilized in this example) while the later indicates those of the subject *i* when not received the treatment. They defined the causal effect of the treatment for subject *i* ($\tau_{i}$) as

(2) $$\begin{array}{c} \tau_{i}=y_{i}\left(z_{i}=1\right)-y_{i}\left(z_{i}=0\right) \end{array}$$

In other words, the fertilizer is said to have a positive causal effect when the crop size of the subject *i* would have been larger *if* subject *i* was treated with the fertilizer, though not treated in reality (and *vice versa*). It, in practice, cannot be observed since only one of the $y_{i}\left(z_{i}=1\right)$ or $y_{i}\left(z_{i}=0\right)$ is observed. However, Rosenbaum and Rubin (1983) showed that an averaged causal effect can be estimated at a specified population level, by using the propensity score.

To illustrate the role of the propensity score, see the example of fertilizer again. If an RCT was conducted, then the probability of the treatment group is unrelated to X, and thus

(3) $$\begin{array}{c} e_{i}=\mathrm{Pr}\left(z_{i}=1|x_{i}\right)=\mathrm{Pr}\left(z_{i}=0|x_{i}\right) \end{array}$$

for all i=1,2,…N. In this case, the average effect of a fertilizer is estimated by

(4) $$\begin{array}{c} E\left[Y|Z=1\right]-E\left[Y|Z=0\right] \end{array}$$

This is a comparison of the expected yield size between the treated group and the untreated group. When, however, RCT was not conducted, $\mathrm{Pr}\left(z_{i}=1|x_{i}\right)\neq \mathrm{Pr}\left(z_{i}=0|x_{i}\right),$ and a simple comparison does not necessarily indicate the causal effect (Rosenbaum and Rubin 1983). For example, if the treatment group tends to receive higher solar radiation ($X;\mathrm{Pr}\left(z_{i}=1|x_{i}=10\right)>\mathrm{Pr}\left(z_{i}=0|x_{i}=1\right)$), which is known to enhance the yield size, we cannot discriminate whether Z or X caused the increase of Y even when.

(5) $$\begin{array}{c} E\left[Y|Z=1\right]>E\left[Y|Z=0\right] \end{array}$$

The propensity score is used to represent how the distribution of X is balanced between the treatment and the untreated group and to adjust the unbalancing by which the result of RCT is emulated. Various propensity score methods have been proposed to estimate the causal effect depending on the target population.

One straightforward methodology is the matching estimator (Rosenbaum and Rubin 1983; Rosenbaum 2002; see also Kluender et al. 2024 for an application in ecology). In this method, pairs of a treated and an untreated subject that have similar propensity scores were constructed and then the outcome of the treated and the untreated groups were compared within each pair. By matching with a subject that has a similar propensity score, differences in confounding factors are adjusted to estimate the causal effect (Imbens 2004). A disadvantage of the matching estimator is that it could lower the statistical efficiency (Abadie and Imbens 2006). If the distribution of X is quite different between $z_{i}=1$ and $z_{i}=0$, there could be no pair to be matched and discarded from the dataset, reducing the sample size. The construction of pairs is not so trivial and has been a matter of controversy among statisticians (Austin 2011, 2014). R packages for the matching estimator are available (e.g., {MatchIt}).

Another commonly used method to estimate causal effects in the potential outcome framework is the propensity score weighted estimator, including the inverse‐probability weighted estimator (IPW), the doubly robust estimator, and the overlap weighted estimator (OW). The IPW estimator, for example, is defined as:

(6) $$\begin{array}{c} \hat{\mathrm{IPW}}=\sum_{i=1}^{N}\frac{z_{i}y_{i}}{e_{i}}+\sum_{i=1}^{N}\frac{\left(1-z_{i}\right)y_{i}}{\left(1-e_{i}\right)} \end{array}$$

This formula reweights each observation by the inverse of the probability of receiving the treatment actually observed. An intuitive idea is that, even when the treatment group *tends* to receive higher solar radiation, we could estimate the causal effect of the fertilizer if there is a subject that was treated but received lower solar radiation. These subjects are assumed to have more information than the treated and higher solar radiation‐received subjects. The weighted estimators infer the causal effect by giving the “importance” of a subject according to the propensity score: The information contributed by Y of untreated subjects with a higher propensity score and treated subjects with a lower propensity score is given greater weight than that of other subjects. The IPW estimator can be more efficient than the matching estimator since it does not require pairs of subjects. But it is still possible to lose statistical efficiency if the distribution of X is quite different between $z_{i}=1$ and $z_{i}=0$ because only a few subjects with a large weight could dominate the result if the propensity score is very large or very small. The overlap weighted estimator (OW) have developed to handle these situations (Li et al. 2018). The OW estimator is defined as:

(7) $$\begin{array}{c} \hat{\mathrm{OW}}=\sum_{i=1}^{N}\left(1-e_{i}\right)z_{i}y_{i}+\sum_{i=1}^{N}e_{i}\left(1-z_{i}\right)y_{i} \end{array}$$

It assigns weights to subjects in proportion to their probability of being assigned to either treatment group. This reduces sensitivity to extreme weights and improves covariate balance because, in contrast to IPW, weighting term $e_{i}$ does not appear in denominator. R packages for these weighted estimators are also available (e.g., {PSweight}, {WeightIt}; Zhou et al. 2025; Greifer 2025).

Other propensity score methods are also known. Covariate adjusting regression analysis with a propensity score considers a regression model where the objective variable is Y and the explanatory variable is Z and e (Austin 2011; Schafer and Kang 2008). This regression approach achieves a consistent estimate of causal effect as long as we specify a correct model between the outcome and the propensity score. The stratification estimator divides the dataset into five or six groups to construct a sub‐dataset that has similar propensity scores within them and combines the differences in the outcome between the treated and untreated groups. Although there are many applications of these methods in medical research, we do not go into further detail here in order to focus on weighted estimators. Interested readers may refer to Austin (2011), for example.

Among the various perspectives to select which propensity score estimators to apply, the target population of a causal effect (i.e., “estimand”) plays a crucial role. The most common estimands are an average treatment effect in the treated (ATT), an average treatment effect in the untreated (ATU), an average treatment effect in the population (ATE), and an average treatment effect in the overlap (ATO). ATT refers to the causal effect of a sub‐population as $E\left[Y\left(Z=1\right)-Y\left(Z=0\right)|Z=1\right]$. In other words, for those who *actually* received the treatment, how would the outcome have differed if they did not receive the treatment. For example, if a conservation or management plan has already been implemented in certain sites, ATT helps assess what would happen if the policy were discontinued in those same sites. This would be especially relevant when funding or administrative capacity is being reconsidered. ATT is also useful for evaluating the impact of potentially harmful exposures such as the effect of light pollution to avian species. Conversely, ATU ($E\left[Y\left(Z=1\right)-Y\left(Z=0\right)|Z=0\right]$) can be useful when considering the potential benefits of extending a policy or treatment to new sites. The most common matching estimator, 1:1 matching are designed to estimate ATT and ATU (e.g., Stuart 2010). ATE refers to the causal effect of the entire population as $E\left[Y\left(Z=1\right)-Y\left(Z=0\right)\right]$, where, unlike ATT or ATU, the target population is not conditional on Z=1 nor Z=0. That is, ATE is defined as the difference between two hypothetical outcomes; if *all* the subjects received the treatment versus *all* the subjects were not received. ATE is well suited for evaluating policies that are intended to be uniformly implemented across a region, for example. The IPW method is commonly used to estimate ATE (e.g., Austin 2023). ATO represents the average treatment effect in the overlap population, which consists of subjects with a similar probability of receiving either treatment or control (Li et al. 2018). That is, ATO evaluates the expected difference in outcomes if each subject in this group had received the opposite treatment—that is, the outcome under control for treated subjects, and the outcome under treatment for untreated subjects. While ATT, ATU, and ATE correspond to a clearly defined populations, the target group for ATO cannot be expressed with a simple conditioning formula. ATO is particularly valuable when treatment decisions involve trade‐offs between costs and benefits. For instance, when benefits clearly surpass costs (e.g., very high or low benefit‐to‐cost ratios), a decision maker of a management plan would introduce the plan without any hesitations, yielding larger propensity score. In contrast, if the costs and benefits are in conflict, the expected effect of the plan needs to be carefully examined in order to decide whether it should be implemented. ATO is suited to handle these situations, and its relationship with the matching estimator (Li et al. 2018; Iacus et al. 2019) and RCT (Thomas et al. 2020) is also discussed. ATO is estimated by the overlapping weighted estimator (Li et al. 2018). See Austin (2023) for details about the choice of estimands.

Several characteristics of the propensity score causal inference are particularly suited for applied ecology. First, it is robust to model misspecification. In contrast to SEM, the propensity score approach does not require the specification of a causal relationship between X and *Y*. Second, the usual propensity score methodology focuses on a case where Z is binary, though extensions to multiple treatment cases are also proposed (reviewed in McCaffrey et al. 2013). These characteristics enable us to estimate the causal effect of an adopted policy (e.g., when a management plan is introduced at some sites but not at random) without detailed knowledge about other confounding factors.

## 3 Methods

### 3.1 Simulation Study

We conducted a simulation study to evaluate the effectiveness of propensity score weighting methods and compared their performance with predictive approaches (i.e., AIC‐based model selection and LASSO estimator).

We generated simulated data consisting of three types of variables: confounding variables (X), treatment variable (Z), and the outcome variable (Y). We applied four types of estimators to estimate the effect of treatment variable on the outcome. We then calculated the bias from the true causal effect for these estimators and counted the number of confidence intervals that correctly included the true value. The simulation framework used in this study is largely based on that of Ramsey et al. (2019), with some important modifications. While Ramsey et al. (2019) compared the performance of different propensity score estimators, our focus is on contrasting propensity score methods with prediction‐oriented approaches such as AIC and LASSO. In addition, we included the overlap weights (OW) estimator, which was not considered in their study. We also tested different strengths of confounding ($\beta_{\text{prop}}$ as defined below).

Data generating process was assumed to be

(8) $$\begin{array}{c} X1,X2,\ldots ,X6\sim N\left(0,1\right) \end{array}$$

(9) $$\begin{array}{c} \mathrm{Pr}\left(Z_{i}=1\right)=p_{i} \end{array}$$

where

(10) $$\begin{array}{c} \log\frac{1-p_{i}}{p_{i}}=X_{i}\beta_{\text{prop}} \end{array}$$

(11) $$\begin{array}{c} Y={\mathrm{Z\beta}}_{\text{cause}}+X\beta_{\text{out}}+N\left(0,1\right) \end{array}$$

Here, $N\left(\mu ,\;\sigma \right)$ is a normal distribution with the mean μ and the standard deviation σ and $\beta_{\text{prop}}={\left(0,0,b,b,-b,-b\right)}^{T},\beta_{\text{out}}$ and $\beta_{\text{cause}}$ are coefficient values for propensity score, the effect of confounders on the outcome and the causal effect of the treatment variable, respectively.

Applied estimators for these data are two propensity score causal inference methods and predictive methods that have been widely applied among ecologists: (a) Inverse‐Probability Weighted Estimator (IPW), (b) Overlapping Weighted Estimator (OW), (c) maximum likelihood estimate of a linear regression model with the smallest AIC score among all the candidate models of seven explanatory variables, (d) LASSO estimate that maximizes the likelihood with the L1 penalty of the coefficient values. For each method, we obtained the point estimate of the causal effect of *Z* on *Y* and its 95% confidence interval. For (a) and (b), the propensity scores were estimated using logistic regression with *Z* as the response variable and X1 to X6 as predictors. We used bootstrap resampling (1000 replications) to estimate standard errors and construct the 95% confidence intervals. For (d), the strength of the L1 penalty was selected using 10‐fold cross‐validation, and the same bootstrap procedure was used.

We tested five different values for the parameters ($b\in \left\{0.5,1.0,1,5,2.0,2.5\right\}$) and three different data size ($N\in \left\{50,100,200\right\}$). $\beta_{\text{out}}$ was fixed to, $\beta_{\text{out}}={\left(1,0.9,0.9,-0.25,-0.25,0,0\right)}^{T}$. Although we tried different values of $\beta_{\text{cause}}$, we report the results of only $\beta_{\text{cause}}=1.0$ as the overall trends were consistent across values. The experiments are repeated in 10,000 trials for each 4 × 3 = 12 setting. All the analysis was conducted in the free software R (ver. 4.3) with packages {Amelia} and {PSweight}.

### 3.2 Application to Real Data

#### 3.2.1 Data Collections

We used original data that was analyzed by Uchida et al. (2021) and rearranged it to compare the differences in statistical results. In summary, Uchida et al. (2021) conducted field observations of Eurasian red squirrel ( *Sciurus vulgaris* ) behavior in 12 urban parks of Obihiro City, Hokkaido, Japan (42°55′ 26.3″ N 143°11′46.1″ E) during 2018 to 2019. Two escape behaviors that were used as objective variables (flight initiation distance; FID and vertical escape distance; VED) were measured in the field. The observer approached a focal squirrel on the ground for foraging at a constant speed and measured the distance between the observer and the squirrel when a target squirrel initiated escape. After measuring FID, the observer continued approaching until the bottom of the trees that squirrels climbed for escaping. The VEDs were measured at the height where a squirrel stopped climbing and gazed at the observer for more than 3 s (Uchida et al. 2017). During the behavioral measurement and using a geographical information system, the observer also recorded environmental variables within the park and around the parks that potentially influence squirrels' tolerance and included them as explanatory variables in the statistical models. Among these explanatory variables, the Feeding variable (= 1, if feeding was found in a park and otherwise 0) is the central focus of interest in this study because controlling humans' feeding activity is the most straightforward action for wildlife management to modulate wildlife tolerance and thus human‐wildlife interactions. We assumed that the number of human visitations in the parks during 30 min of field observation (NH), the number of trees in the parks (TN), the total pathway length in the parks (PL), human population density around the park (P), and the proportion of green space cover in the parks (PGS) could be confounding variables since these variables are known to be associated with wildlife's tolerance to humans. Details of each variable in the model are explained in Table 1. More detailed information on study sites and field observations is explained by Uchida et al. (2021).

**TABLE 1:** Summary of the collected variables for FID and VED. The table indicates whether each variable was considered a confounder (✓)—a variable that may influence both the outcome (FID or VED) and the occurrence of artificial feeding in each park—or not (✗).

Variables	Details	Confounding
TD	Tree density in park	✗
DT	Distance from tree	✗
CO	Local canopy cover	✗
NH	Number of human visitations	✓
TN	Local tree density	✓
PL	Pass way length	✗
NP	Density of recreational equipment	✗
PGS	Proportion of green space in park	✓
RG	Proportion of green space around the park	✗
*P*	Population density around the park	✓

#### 3.2.2 Data Preparation

We preprocessed the collected data before conducting further statistical analysis. Following a common practice in ecology (Schielzeth 2010), all continuous variables were standardized to have a mean of 0 and a standard deviation of 1 to enhance interpretability. This standardization is particularly important when applying LASSO, as its penalty term is based on the absolute magnitude of regression coefficients. Since some variables contain missing observations, we conducted the MCAR hypothesis test to check whether the data is missing completely at random (MCAR) or not (Little 1988). The null hypothesis was not rejected (*p* = 0.067). We then applied the multiple imputation methodology to complement these values and obtained 1000 pseudo‐complete datasets.

#### 3.2.3 Statistical Analysis

We applied two statistical approaches to the same dataset and compared the results. The first one is predictive approaches based on AIC and LASSO estimators. We constructed a linear regression model, where FID or VED is the objective variable and Feeding, TD, DT, CO, NH, TN, PL, NP, PGS, RG, and P are the explanatory variables. We then conducted variable selection based on AIC and LASSO. We explored all the candidate models with different combinations of explanatory variables and selected the lowest AIC model. For the strength of the LASSO penalty, we chose the optimal value that minimizes the cross‐validation error, and the standard error was obtained by bootstrapping (50 replications). The coefficient of the Feeding variable was extracted from the best model of both AIC and LASSO.

In the second approach, the causal inference approach based on a propensity score was analyzed. We calculated the propensity score using the logistic regression model, where NH, TN, PGS, and P were used for the confounding variable. We checked the standardized mean differences of the confounding variables to ensure the propensity score successfully adjusted the imbalance between the treated and the untreated group. We then estimated the causal effect of the Feeding variable on FID and VED by the overlap weighing estimator (OW). We also included the remaining explanatory variables, DT, TD, CO, PL, NP, and RG, assuming it could also influence FID and VED but not on the Feeding. That is, for i=1,2,…,N,

(12) $$\begin{array}{c} {\text{Feed}}_{i}\sim {\mathrm{NH}}_{i}+{\mathrm{TN}}_{i}+{\mathrm{PGS}}_{i}+P_{i} \end{array}$$

(13) $$\begin{array}{c} {\mathrm{FID}}_{i}\sim {\text{Feed}}_{i}+{\mathrm{DT}}_{i}+{\mathrm{TD}}_{i}+{\mathrm{CO}}_{i}+{\mathrm{PL}}_{i}+{\mathrm{NP}}_{i}+{\mathrm{RG}}_{i} \end{array}$$

(14) $$\begin{array}{c} {\mathrm{VED}}_{i}\sim {\text{Feed}}_{i}+{\mathrm{DT}}_{i}+{\mathrm{TD}}_{i}+{\mathrm{CO}}_{i}+{\mathrm{PL}}_{i}+{\mathrm{NP}}_{i}+{\mathrm{RG}}_{i} \end{array}$$

where (12) is a logistic regression model assuming a Bernoulli distribution, used to estimate the propensity score. Models (13) and (14) are weighted linear regression models that evaluate the effect of the Feed variable on FID and VED, respectively. Each observation is weight by $\left(1-e_{i}\right)$ if ${\text{Feed}}_{i}=1$ and by $e_{i}$ otherwise, where $e_{i}$ is the estimated propensity score obtained by (12).

For the evaluation of the point estimate, we applied the bootstrap resampling (50 replications) by which the standard error of the estimator is obtained to calculate the 95% confidence interval for both the predictive and the causal inference approach. The above methods were applied to all the 1000 pseudo‐complete datasets, and we combined these results by the rule of Rubin (1987) to calculate the overall estimate and its standard error. All the analysis was conducted in the free software R (ver. 4.3) with packages {Amelia} and {PSweight}.

## 4 Results

### 4.1 Simulation Studies

Table 2 shows the results of the point estimates. For the propensity score methods (IPW and OW), the mean of the 10,000 estimates was almost equal to the true causal effect, indicating that these methods are unbiased estimators. In contrast, the mean estimates obtained from AIC and LASSO were biased relative to the true causal effect, when the sample size N was small or the strength of confounding b was large. These results indicate that AIC and LASO may yield misleading results in causal inference. Although the bias of AIC estimator tends to be smaller as the sample size increases, the bias of LASSO remains relatively large even for larger samples, indicating that the application of LASSO requires extra caution in practice.

**TABLE 2:** Comparisons of bias between the four types of estimators. The average of 10,000 point estimate is reported for different sample sizes ($N\in \left\{50,100,200\right\}$) and for different strengths of the confounding ($b\in \left\{0.5,1.0,1,5,2.0,2.5\right\}$). AIC and LASSO tend to underestimate the impact of the treatment variable, while the propensity score methods achieved unbiased estimates.

Sample size	IPW	OW	AIC	LASSO
*b* = 0.5				
*N* = 50	1.00	1.00	0.94	0.84
*N* = 100	1.00	1.00	0.99	0.92
*N* = 200	1.00	1.00	1.00	0.95
*b* = 1.0				
*N* = 50	1.00	1.00	0.90	0.79
*N* = 100	1.00	1.00	0.97	0.89
*N* = 200	1.00	1.00	1.00	0.94
*b* = 1.5				
*N* = 50	1.00	1.01	0.87	0.76
*N* = 100	1.00	1.00	0.96	0.87
*N* = 200	1.00	1.00	0.99	0.92
*b* = 2.5				
*N* = 50	1.00	1.00	0.84	0.74
*N* = 100	1.00	1.00	0.94	0.85
*N* = 200	1.00	1.00	0.99	0.92

Table 3 shows the coverage rates of the 95% confidence intervals. For the propensity score methods, the empirical coverage rates were close to the nominal 95%, indicating that these methods provide reliable uncertainty estimates. In contrast, AIC and LASSO showed lower coverage rates, further suggesting that they may be inappropriate for causal inference tasks. In addition, while the point estimate of AIC reaches the true value as the sample size grows, the performance of coverage rates does not improve much.

**TABLE 3:** Comparisons of coverage rate between the four types of estimators. The proportion of a 95% confidence interval that covers the true value is reported for different sample sizes and for different strengths of the confounding. AIC and LASSO tend to be too optimistic about the uncertainty of the interval estimate, while the propensity score methods keep their nominal rate.

Sample size	IPW	OW	AIC	LASSO
*b* = 0.5				
*N* = 50	0.9753	0.9567	0.8924	0.3755
*N* = 100	0.9574	0.9503	0.9304	0.1611
*N* = 200	0.9505	0.9472	0.9427	0.019
*b* = 1.0				
*N* = 50	0.9808	0.9677	0.8356	0.3904
*N* = 100	0.959	0.9437	0.8986	0.2071
*N* = 200	0.9535	0.9475	0.9304	0.0447
*b* = 1.5				
*N* = 50	0.9808	0.9735	0.7891	0.3969
*N* = 100	0.9641	0.9482	0.8732	0.2406
*N* = 200	0.9484	0.9407	0.9139	0.0656
*b* = 2.0				
*N* = 50	0.9772	0.9747	0.7587	0.3907
*N* = 100	0.9632	0.9538	0.8585	0.252
*N* = 200	0.9467	0.9407	0.9077	0.0788

### 4.2 Application to Real Data

**FIGURE 1:**
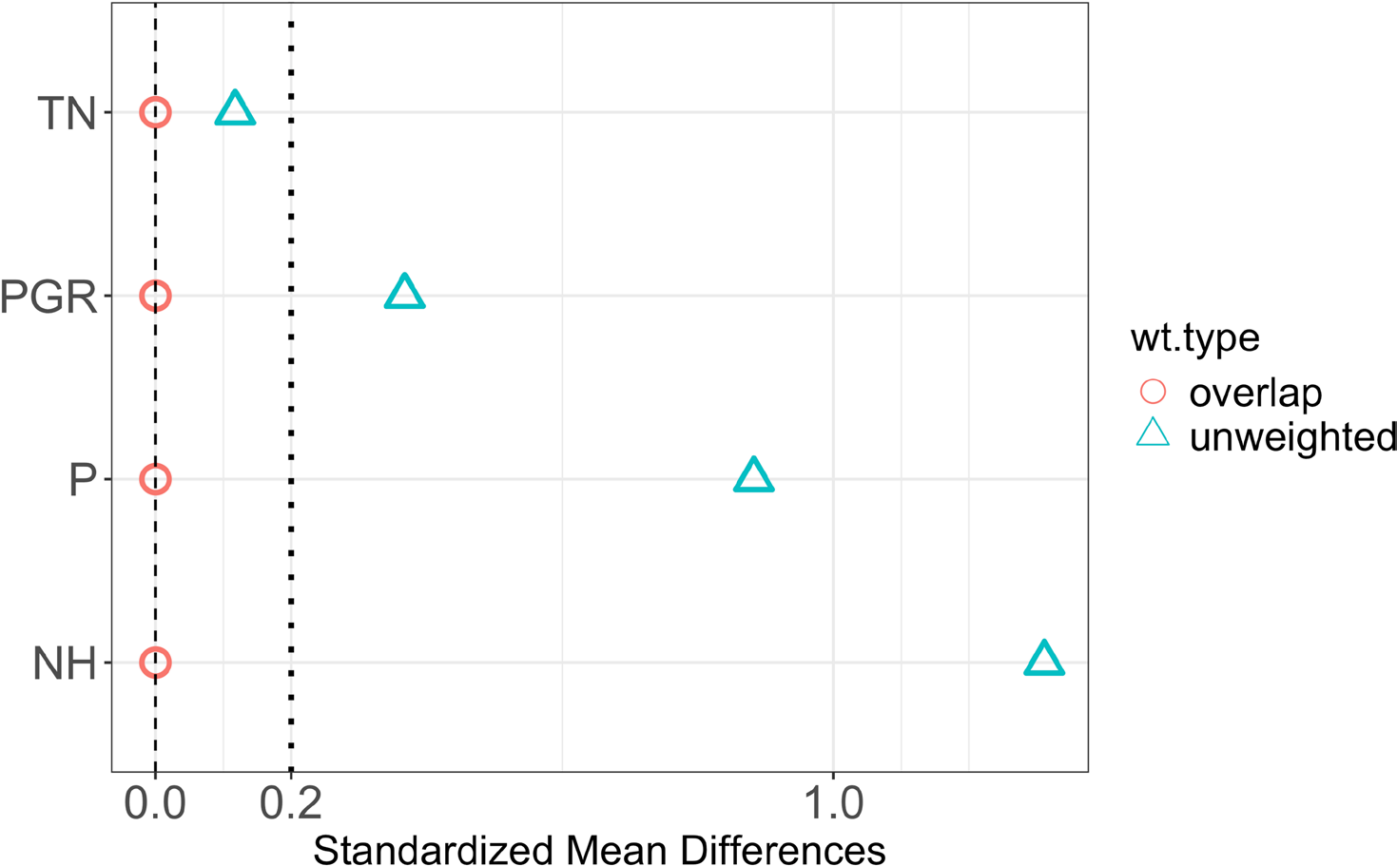
Comparison of the standardized mean differences (SMD) of confounding variables between the unweighted and the overlap‐weighted data. SMD quantify the difference in means of each covariate between treatment groups, scaled by the pooled standard deviation; smaller SMD values indicate better balance between groups. Triangles represent the SMD values before weighting (unweighted), and circles represent those after applying overlap weighting of the propensity score. The dashed vertical lines at 0.0 and 0.2 indicate reference values: An SMD below 0.2 is generally interpreted as an acceptable level of covariate balance. In the unweighted data, covariate distributions differ between parks with and without artificial feeding, which would lead to a biased estimate of the causal effect if outcomes (FID and VED) were compared directly. After applying overlap weighting, the differences in covariate means are substantially reduced, indicating improved balance and supporting a less biased estimation of the treatment effect.

We found that the difference in confounding distribution is properly corrected by the overlapping weighting of the propensity score (Figure 1, the standardized mean differences were less than 0.01 for all the confounding variables). For the point estimate, the OW estimator revealed a larger absolute effect of the Feeding variable (−1.12 for FID and −1.07 for VED) than the predictive approaches (AIC: −1.00, LASSO: −0.79 for FID and AIC: −0.57, LASSO: −0.44 for VED). The 95% confidence interval of OW was [−0.18, −2.07] for FID and [0.26, −2.19] for VED, while those of the predictive approaches were (AIC: [0.05, −2.05], LASSO: [0.22, −1.89] for FID and AIC: [0.41, −1.56], LASSO: [0.44, −1.33] for VED).

## 5 Discussion

In this paper, we introduced causal inference methods based on propensity score and compared their performance with commonly used statistical methods that aim to improve predictive accuracy. We also, as a case study, applied a propensity score method to FID and VED, behavioral traits of the wild Eurasian red squirrel, *Sciurus vulgaris*, and evaluated the causal effect of artificial feeding. By simulation experiments, we found that, while the best AIC model and LASSO estimate tend to yield misleading results with biased point estimates and overly optimistic confidence intervals, propensity score methods are reliable estimators to evaluate causal effects because they are unbiased estimators with valid confidence intervals.

### 5.1 Statistical Performance of the Propensity Score Methods

The biases inherent in the best AIC model and LASSO estimate raise concerns about the validity of previous evidence for the effectiveness of conservation and management plans. Our application to the real data, for instance, revealed that the effect of artificial feeding on FID and VED may be greater than previously known, indicating that the previous report could be slightly misleading. While Uchida et al. (2021) applied LASSO‐based statistical inference to assess the effect of artificial feeding on these traits, our propensity score analysis yielded a larger absolute point estimate compared to those from AIC and LASSO. For example, the absolute standardized coefficient of artificial feeding on FID increased by 12% compared to AIC and 41% compared to LASSO. Furthermore, we found a statistically “significant” effect of artificial feeding on FID (i.e., the 95% confidence interval did not include zero), whereas AIC and LASSO failed to detect this effect. Such differences could give important insights for park management, as a stronger effect on FID, which represents wildlife tolerance toward humans, could alter policymaking for artificial feeding (see below for detailed discussion). These findings are also consistent with our simulation experiments: on average, AIC and LASSO yield biased estimates of the causal effect, and their 95% confidence intervals are often unreliable. In particular, the coverage rate for a “significant” variable selected by the best AIC model can drop to 75%, and to just 2% for LASSO (Table 3). Increasing the sample size does not resolve this issue for LASSO. While the accurate estimate of the causal impact serves a fundamental role in evaluating these practices, conservation and management plans, further scrutiny of previous studies may reveal other cases where the impact of a conservation or management plan is misleadingly reported due to the use of prediction‐oriented methods like AIC and LASSO. In contrast, propensity score methods may offer more reliable estimates by focusing explicitly on causal inference rather than prediction.

### 5.2 Potential Implications to Wildlife Conservation and Management

Propensity score methods have broad applicability to a wide range of applied ecology by offering a framework to identify key drivers that are associated with biodiversity conservation and wildlife management issues. For example, wildlife's behaviors and population demography are influenced by a variety of factors (e.g., human factors and non‐human ecological factors), which makes it harder to clearly detect the particular drivers. Although the approach of analyzing correlations between variables—a major approach of field ecologists—has been largely used in observational data collected in the field, identifying causal relationships is often challenging. This may hinder the development of concrete management strategies and the implementation of prompt action. Applying propensity score methods may enable us to quantify the effect of targeted factors by statistically controlling for confounding covariates. Propensity score analysis is considered particularly effective in urgent situations in which experimental verification is not feasible.

More specifically, our results provide reliable information to support park managers in making decisions about whether to allow artificial feeding as a means of managing human–squirrel interactions in urban environments. The OW estimate indicates that the effect of artificial feeding on FID and VED may be stronger than previously thought. Since FID and VED are widely recognized as indicators of wildlife tolerance toward humans, our findings suggest that managing artificial feeding practices could contribute to managing human–squirrel interactions as part of a broader strategy. For people resident in urban areas, interactions with wildlife may have benefits by enhancing cultural services and so our quality of life (Soga and Gaston 2020) and by offering opportunities for environmental education (Uchida et al. 2021). These benefits would be mitigated or even canceled by negative aspects of increased tolerance to humans, including disease transmission, human injury, and increased vulnerability to real predators (Uchida et al. 2023; Geffroy et al. 2015). Indeed, the Hokkaido government has taken a position against feeding wild animals (https://www.pref.hokkaido.lg.jp/ks/skn/environ/parks/tyui‐yasei1.html). Controlling artificial feeding can be an option to achieve a good balance between benefits and costs. It is important to note that, in making decisions about artificial feeding, park managers need to consider additional factors. For example, energetic or fitness costs on animals caused by human disturbance are context dependent (Gill et al. 1996; Sutherland 2007; Bisson et al. 2009), and the financial and human resources required to implement management plans may affect the feasibility of intervention, as well as the benefits of restricting artificial feeding. Despite these complexities, however, assessing the effect of artificial feeding remains fundamental. Commonly used methods such as AIC‐based model selection and LASSO can yield biased estimates and overly narrow confidence intervals, potentially misleading decision makers. Therefore, methods that provide more reliable causal inference—such as propensity score approaches—are essential for wildlife management.

### 5.3 Limitations of Propensity Score Methods for Future Implementations

The propensity score methods could have several limitations when applied to broader cases because the validity of the estimated causal effect depends on a few assumptions. The key idea behind the propensity score methods is that the causal effect of a treatment variable (i.e., presence of feeding in our case) can be estimated by adjusting differences of confounding variables between the treated and the non‐treated group, where the propensity score acts as a summary statistic that represents these differences: by providing “importance” for observations that contribute to a fair comparison between the treated and the non‐treated group, propensity score weighted estimators achieve a consistent estimate of the true causal effect. Thus, adjustment can be useless if the distributions of confounding variables are quite dissimilar between the treated and the non‐treated group, since the result depends on only a few observations that have very large propensity scores. This can happen when confounding variables are strongly correlated to the treatment variable and the distribution of the propensity score has no or very little overlap (e.g., a case where most of the propensity score is nearly 1 for the treatment group and 0 for the non‐treatment group) (Crump et al. 2009). Indeed, it is natural, for example, for a wildlife manager to decide whether a conservation plan should be introduced or not, considering various environmental factors. Even though several statistical studies have shown that the overlapping weighing estimator, a method we applied, is more robust to the difference of the propensity score distribution than previously known estimators typified by the IPW estimator or matching estimator (Li et al. 2018, 2019), its performance should be better evaluated by future studies. Ramsey et al. (2019) discussed that the concept of the propensity score is useful for designing observational studies, as well as data analysis. Establishing data collection designs for the ecology that ensure the better overlapping of propensity scores would be an essential perspective for the future application of propensity score methods. Understanding the theoretical requirements of the propensity score methods and their validity in actual data would be beneficial to ensure the reliability of the estimated causal effect.

The use of the causal inference method requires extra care because the term “causality” is ambiguous and even confusing in some cases. Since Scottish philosopher David Hume examined this concept in 1739 (Hume 2000), many definitions have been proposed to characterize causality (probabilistic theory of Reichenbach 1956; Suppes 1970; INUS theory of Mackie 1965; counterfactual theory of Lewis 1973; intervention theory of Spirtes et al. 2000 to name just a few) and are still the focus of many studies for philosophers. Beyond the philosophical community, the empirical dynamic modeling approach is increasingly applied in ecology to time‐series data (Sugihara et al. 2012), yet its statistical justifications have not been known (e.g., bias, consistency, or standard error), at least so far. Although many techniques have been proposed to find causality, differences in the causal concept should be noted as well as these scopes, theoretical justification, and limitations.

## Author Contributions

**Yusaku Ohkubo:** conceptualization (lead), formal analysis (lead), methodology (lead), software (equal), supervision (lead), writing – original draft (lead). **Ozora Takeda:** data curation (equal), formal analysis (equal), writing – original draft (supporting). **Kenta Uchida:** data curation (lead), investigation (lead), resources (lead), writing – original draft (equal).

## Conflicts of Interest

The authors declare no conflicts of interest.

## Data Availability

Data and R scripts are available from Dryad repository for review: https://doi.org/10.5061/dryad.wstqjq2xm.
